# Molecular Characterization of Ancient Prosaposin-like Proteins from the Protist *Dictyostelium discoideum*

**DOI:** 10.1021/acs.biochem.4c00479

**Published:** 2024-10-18

**Authors:** Marius Ortjohann, Matthias Leippe

**Affiliations:** Comparative Immunobiology, Zoological Institute, Christian-Albrechts-Universität Kiel, Am Botanischen Garten 1-9, D-24118 Kiel, Germany

## Abstract

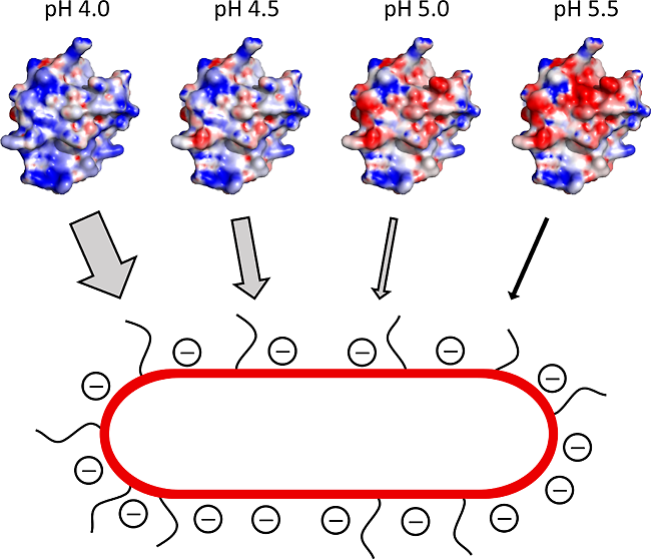

To combat the permanent exposure to potential pathogens every organism relies on an immune system. Important factors in innate immunity are antimicrobial peptides (AMPs) that are structurally highly diverse. Some AMPs are known to belong to the saposin-like proteins (SAPLIPs), a group of polypeptides with a broad functional spectrum. The model organism *Dictyostelium discoideum* possesses a remarkably large arsenal of potential SAPLIPs, which are termed amoebapore-like peptides (Apls), but the knowledge about these proteins is very limited. Here, we report about the biochemical characterization of AplE1, AplE2, AplK1, and AplK2, which are derived from the two precursor proteins AplE and AplK, thereby resembling prosaposins of vertebrates. We produced these Apls as recombinant polypeptides in *Escherichia coli* using a self-splicing intein to remove an affinity tag used for purification. All recombinant Apls exhibited pore-forming activity in a pH-dependent manner, as evidenced by liposome depolarization, showing higher activities the more acidic the setting was. Lipid preference was detected for negatively charged phospholipids and in particular for cardiolipin. Antimicrobial activity against various bacteria was found to be inferior in classical microdilution assays. However, all of the Apls studied permeabilized the cytoplasmic membrane of live *Bacillus subtilis*. Collectively, we assume that the selected Apls interact by their cationic charge with negatively charged bacterial membranes in acidic environments such as phagolysosomes and eventually lyse the target cells by pore formation.

## 1 Introduction

The abundance of potential pathogens requires the presence of an effective immune system in all organisms. In vertebrates, this function is fulfilled by an innate and an acquired component, whereas invertebrates entirely rely on innate immunity. An important part of the molecular arsenal of innate immunity are antimicrobial peptides (AMPs) that inhibit the growth or kill invading pathogens.^1^ AMPs are a very diverse group of peptides, some of which are described to belong to the saposin-like proteins (SAPLIPs). These proteins contain a saposin B-domain, the respective fold of which is characterized by four to five α-helices interconnected by mostly three disulfide bonds.^2^

SAPLIPs have been analyzed in various organisms and have been shown to have very different functions.^3,4^ For example, the name-giving saposins are not described as AMPs, but as proteins involved in sphingolipid degradation.^5^ The best known and most intensely studied antimicrobial SAPLIP members are the porcine NK-lysin and the human granulysin, polypeptides that are active against a broad spectrum of microorganisms, not only acting toward bacteria but also toward intracellular parasites and fungi.^6−8^ Moreover, SAPLIPs have also been considered to be pathogenicity factors of parasitic amoebae as they are pore-forming proteins, for example in *Entamoeba histolytica* or *Naegleria fowleri*; the corresponding proteins were named “amoebapores” and “naegleriapores”, respectively.^9−12^ In the nematode *Caenorhabditis elegans* similar pore-forming proteins were characterized and termed “caenopores”. These proteins apparently defend the worm against potential pathogens and are instrumental for growth on *Escherichia coli* food bacteria.^13−15^ Notably, the solution structures of various antimicrobial SAPLIPs, e.g. NK-lysin, granulysin, amoebapore A, and caenopore 5, have been solved, and mechanisms of membrane permeabilization have been proposed that include membrane perturbation or the formation of well-defined pores in target cell membranes.^16−19^

Recently, SAPLIP-encoding genes have also been identified in the genome of the soil-dwelling social amoeba *Dictyostelium discoideum*, and named amoebapore-like peptides (Apls).^20^ The species^21^ has become a model organism for very different research topics including innate immunity, infection biology, phagocytosis, evolution of multicellularity, and neurological disorders.^22−26^*D. discoideum* feeds on bacteria and shows a special behavior regarding its life cycle. In the absence of sufficient food, the amoebae stop their vegetative phase and start aggregation. The aggregates then pass through various multicellular stages including a mobile pseudoplasmodium coined slug, which can migrate along the soil surface. Finally, aggregates form a fruiting body that comprises a stalk built from dead cells and a sorus that contains live spores.^25,27^

Several antimicrobial factors have been identified and characterized in *D. discoideum* that have been shown to contribute to bacteriolytic activity including lysozymes and proteins of the Bad family.^28−30^ Yet, only little is known about the antimicrobial function of Apls. In the *D. discoideum* genome, 17 SAPLIP-encoding genes have been identified, each containing one to five saposin B-domains, giving rise to a total of 33 putative Apls.^20^ Besides the Apl peptides, saposin B-domains can also be identified in acyloxyacyl hydrolase that acts on lipopolysaccharides^31,32^ or in the proteins countin A and countin B that are involved in determination of the correct aggregate size as part of a counting factor complex.^33,34^

Out of the many potential Apls, the in vivo function of AplD and AplA have been investigated, the latter of which is involved in killing of extracellular *Pseudomonas aeruginosa*.^20,32,35^ The in vivo functions of AplJ, AplP, AplN, AplB, and AplH have also been studied but to a very limited extent so far.^20,32,35^ The biochemical properties at the protein level have been determined for the recombinant AplD only.^20^ The comprehensively characterized *aplD* gene is transcribed almost exclusively at the late developmental stages of *D. discoideum*. In accordance, the corresponding polypeptide has been shown to participate in the protection of the multicellular slug stage from infection with *Klebsiella pneumoniae*. Furthermore, AplD appears to be necessary for solid growth on virulent *K. pneumoniae*, all together indicating a function of AplD in immune defense rather than in the digestion of phagocytosed bacteria. Importantly, recombinant AplD forms pores in liposomes and permeabilizes the membranes of live bacteria.

The rich but rather unexplored arsenal of Apls intrigued us to analyze these potential antimicrobial polypeptides further. To focus on a function in immune defense, we were aiming to characterize other Apls that show high transcription primarily in the late developmental stages, at the protein level.

Here, we present the purification and biochemical characterization of four polypeptides comprising a saposin B-domain derived from the precursor proteins AplE and AplK of *D. discoideum* that contain two saposin domains each. AplE1, AplE2, AplK1, and AplK2 were produced as recombinant proteins from *E. coli* using an inducible self-splicing intein to remove the affinity tag used for purification. Antimicrobial activity of purified Apls was monitored with live bacteria and peptides were characterized regarding their pH dependency of activity and their lipid preference using liposomes.

## 2 Materials and Methods

### 2.1 Plasmid Construction

**Figure 1:**
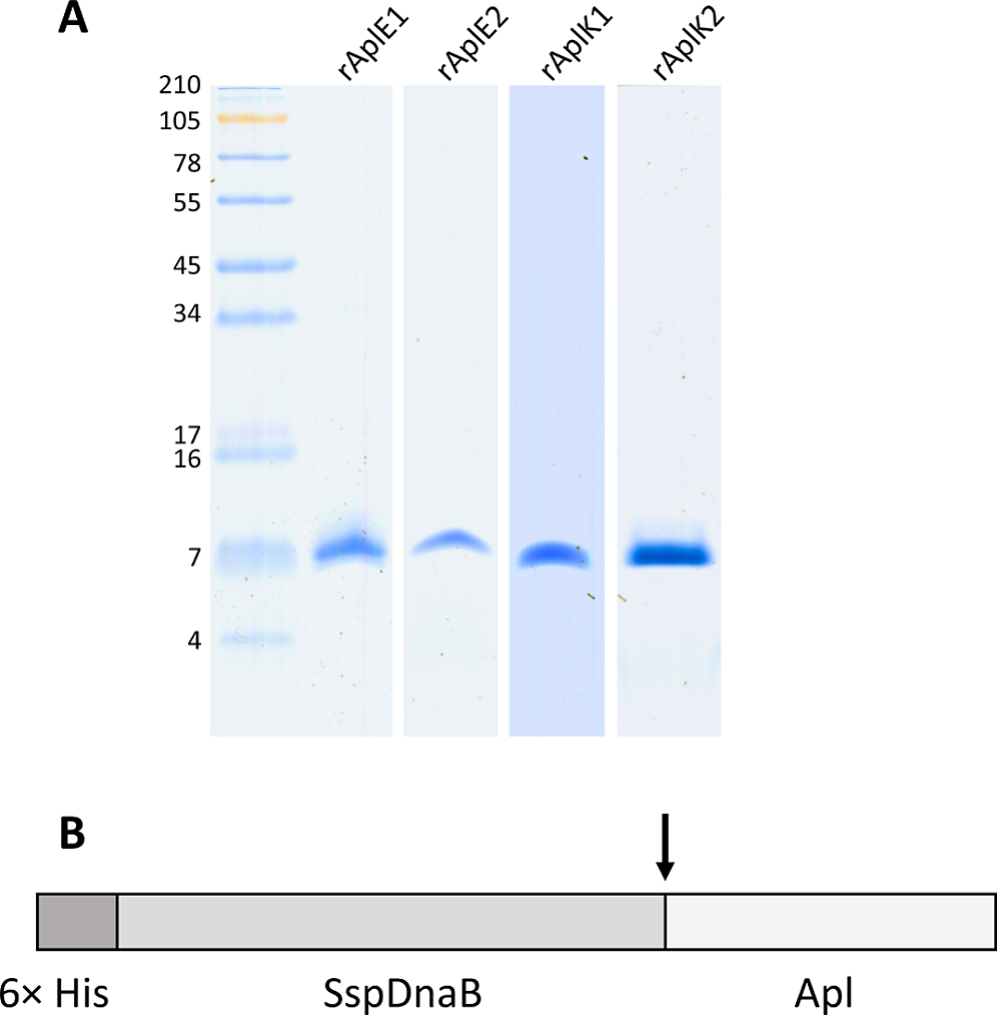
Purified recombinant Apls and schematic representation of the recombinant Apls fused to the intein SspDnaB. (A) Polyacrylamide gels of purified recombinant Apls rAplE1, rAplE2, rAplK1, and rAplK2 stained with Coomassie blue. Molecular masses in kDa of the protein marker are shown on the left. (B) The complete recombinant fusion protein contains the Apl protein (light gray), that is fused to the C-terminus of the intein SspDnaB (mid gray), which contains an N-terminal 6× His-tag (dark gray). The position for intein splicing is marked with an arrow.

The recombinant Apls were overexpressed as fusion proteins with a self-splicing intein. For that purpose the encoding *apl* sequences were purchased as codon optimized genes for overexpression in *E. coli*. At their 5′ ends, these sequences were fused to the intein *sspdnaB* (pTWIN1 vector, New England Biolabs) by using standard polymerase chain reaction techniques, and cloned into the vector pET28a (Novagen) with the restriction enzymes *Nde*I and *Bam*HI. In this way, SspDnaB was fused with a vector-encoded N-terminal 6× His-tag for affinity purification, which resulted in the overexpression of recombinant fusion proteins that are composed of His-tagged SspDnaB fused to the N-terminus of the respective Apl (Figure 1). To enable efficient splicing of SspDnaB an additional glycine residue was inserted at the junction site of intein and Apl, which remains at the N-terminus of the respective Apl after intein splicing. The N- and C-terminus of the recombinant polypeptides were deduced according to the name-giving natural amoebapores of *E. histolytica*, which served as prototypes, and the distribution of charged amino acid residues at the N-terminus. The resulting final recombinant Apl polypeptides are as follows: rAplE1: GVAQCDVCNYLVTMVEVFVEQNRSETYISNSLEKVCEIIPREDYKSTCRSIVLAYTKDIIQLIINREPSEKICQEIKAC; rAplE2: GALQCTICKLVATKLEEYIQSNKTIEEIENELDDFCKIAFEKDPTQCQGFVQQYVPMILSFIKSKEDPTQACIKLKFC; rAplK1: GKANPTECEACQIVIGYVENLVLHSNKTQGEIEKELDKLCNMVSPRYKPTCDSIVSVYTTEIIQLILNKETPDLICKEIKVC; rAplK2: GRSTSECEICQVFVSKLESYISTNKSQEEIMEELDNACDYMKSFEQQCKQMVQDYVPELIEIMSTTEDPNKVCSQISLC (underlined residue G corresponds to the additional glycine residue at the N-terminus of the respective Apl).

### 2.2 Overexpression and Purification

For overexpression, *E. coli* SHufle T7 Express lysY (New England Biolabs)^36^ was transformed with the respective plasmids and grown in Luria–Bertani (LB) medium supplemented with 50 μg/mL kanamycin sulfate for selection. Growth was performed at 28 °C. When cultures reached OD_600_ ∼ 0.8 overexpression was induced by the addition of 1 mM isopropyl-β-d-thiogalacto-pyranoside. Temperature was then lowered to 25 °C and cultures were further incubated for 5 h before cells were harvested by centrifugation.

For protein purification, cells from a 2 L overexpression culture were suspended in 100 mM Tris-HCl, 300 mM NaCl, 5 mM imidazole, pH 8.2. Cells were then lysed by sonification at 60% duty cycle at 40% power for 3 min (Sonoplus HD 2200 sonicator, MS-73 titanium microtip, Bandelin electronic GmbH). Cell debris were removed by centrifugation at 4 °C at 48,000*g* for 30 min. Cleared extract was applied to a 1 mL Ni-NTA column and proteins were eluted by stepwise increasing the concentration of imidazole to 200 mM. To induce the splicing reaction of the intein, the pH value was slightly acidified and the temperature was increased.^37^ For this purpose, fractions containing the fusion protein of intein and Apl were diluted 5-fold with 100 mM Bis-Tris, pH 6.5 and further incubated at 25 °C for 72–120 h. To remove the His-tagged intein after the splicing reaction, the protein solution was adjusted to pH 8.0 by the addition of 2 M Tris solution followed by incubation with 1 mL Ni-NTA resin for 1 h under constant shaking. The Ni-NTA resin was then removed from the protein solution by centrifugation, and the Apl-containing supernatant was dialyzed (cutoff: >3.5 kDa) against a 100-fold volume of 25 mM Tris-HCl, pH 8.2. For further purification, the protein solution was applied to a Ressource Q 6 mL column (Pharmacia) that was equilibrated with 25 mM Tris, pH 8.2 and eluted with a gradient of increasing NaCl concentration in the same buffer. Fractions containing Apl protein were then pooled, diluted 2.5-fold with 0.1% trifluoroacetic acid (TFA), and applied to a Sep-Pak tC18 (reversed-phase) Vac Cartridge (500 mg, Waters) that was equilibrated with 0.1% TFA. Elution of recombinant Apls was performed by stepwise increasing the acetonitrile concentration. After this purification step, recombinant Apls were apparently pure as evidenced by sodium dodecyl sulfate-polyacrylamide gel electrophoresis (SDS-PAGE) analyses. Proteins were subsequently lyophilized and stored at −80 °C until use. Functional assays with recombinant Apls were performed with proteins that were dissolved in 0.01% TFA. Protein concentrations were determined with the bicinchoninic acid assay (Pierce, Thermo Scientific).

### 2.3 Pore-Forming Activity

The liposome depolarization assay^38^ was performed to test the pore-forming activity of the recombinant Apls at pH values between 4.4 and 7.4. The assay was performed with liposomes made from asolectin (AL) as described earlier in detail.^9^ The peptide alamethicin (Sigma-Aldrich) served as a control. In brief, a diffusion potential is induced across the membrane of liposomes by the addition of valinomycin, thereby quenching the fluorescence of a voltage sensitive dye until a pore-forming molecule is added that breaks down this diffusion potential. This dissipation results in an increase of fluorescence that can be monitored. A 5% increase of the fluorescence intensity compared to the intensity before the addition of valinomycin is defined as 1 unit (U). Pore formation was tested at 25 °C in 50 mM Tris maleate, 50 mM K_2_SO_4_, 0.5 mM ethylenediaminetetraacetic acid (EDTA) at pH values between 4.4 and 7.4. The significance of disulfide bonds was tested by reduction and alkylation of cysteine residues. To this end, Apls were alkalized with Tris-HCl, and cysteines were reduced by incubating the sample with 20 mM dithiothreitol at 60 °C for 30 min. Free thiol groups were alkylated by the addition of 100 mM iodoacetamide and incubation of samples at 20 °C for 60 min.

### 2.4 Lipid Preferences

Lipid preference was analyzed using the calcein release assay^39^ with liposomes made from AL or from 1-α-phosphatidylcholine (PC) alone, or from 3:1 (w/w) compositions of PC and either 1-α-phosphatidylglycerol (PG), 1-α-phosphatidylinositol (PI), 1-α-phosphatidylethanolamine (PE), 1-α-phosphatidylserine (PS), cardiolipin (CL), or sphingomyelin (SM) (all lipids were purchased from Avanti Polar Lipids, Inc.). Liposomes were prepared in 10 mM *N*-(2-hydroxyethyl)piperazine-*N*′-ethanesulfonic acid (HEPES), 30 mM calcein, 1 mM EDTA, pH 7.4. To remove untrapped calcein, liposomes were applied on to a NAP-5 size exclusion column, and elution was performed with 10 mM HEPES, 150 mM NaCl, 1 mM EDTA, pH 7.4. Lipid specificities were studied in 50 mM Tris maleate, 50 mM K_2_SO_4_, 0.5 mM EDTA, pH 4.4. In principle, fluorescence of entrapped calcein is quenched at the high concentrations within liposomes. As a consequence, permeabilization of the vesicle membrane results in an increase of fluorescence that can be monitored. Lysis of liposomes is normalized to fluorescence values of 100% liposome lysis, which is obtained by the addition of 0.1% Triton X-100. The peptide alamethicin served as control.

### 2.5 Antibacterial Activity

Antibacterial activity was determined as minimal inhibitory concentration (MIC) and as minimal bactericidal concentration (MBC). The assay was performed with protein concentrations of up to 20 μM in 10 mM sodium phosphate buffer, pH 5.2 at 37 °C. Cell densities of exponentially growing bacteria were adjusted with LB medium (adapted to pH 5.2), and 100 colony forming units (CFU)/well were incubated with serial dilutions of recombinant Apls in 96-well polystyrene plates overnight as described.^40^ Correlation of OD_600_ between the tested bacteria and CFU was established in advance by plating bacterial cultures at different OD_600_ values in different dilutions and by subsequent CFU counting. MIC was determined as the lowest peptide concentration at which no growth could be observed. The MBC was determined by plating out overnight-incubated mixtures that showed no bacterial growth on LB agar plates, and by screening for the lowest peptide concentrations for which no colonies were observed after incubation of these plates at 37 °C. The following bacteria were tested with the four recombinant Apls and with melittin (Sigma-Aldrich) that served as control peptide: the Gram-negative bacteria *E. coli* D31, *K. pneumoniae* ATCC 13883, and *P. aeruginosa* ATCC 10145, and the Gram-positive bacteria *Priestia megaterium* ATCC 14581 (formerly *Bacillus megaterium*), *Staphylococcus aureus* ATCC 12600, and *Bacillus subtilis* ATCC 6051.

### 2.6 Membrane Permeabilization of Live Bacteria

Permeabilization of bacterial cells was analyzed using the SYTOX Green assay.^11,14^ In principle, the fluorescent dye SYTOX Green intercalates into the DNA of permeabilized cells resulting in an increase of fluorescence. The assay was performed in 96-well polystyrene plates in 10 mM sodium phosphate, 25 mM NaCl, pH 5.2 in serial dilutions with the recombinant Apls, and with 1 × 10^6^ CFU/well (*E. coli* D31) or 1 × 10^5^ CFU/well (*B. subtilis* ATCC 6051) from the logarithmic phase. Fluorescence values for maximal cell membrane permeabilization were obtained by incubating the respective cells with a serial dilution of melittin (up to 4 μM).

### 2.7 Bioinformatics

The three-dimensional structure of the recombinant Apls was modeled using the colabfold notebook AlphaFold2_mmseqs2.^41^ The isoelectric points (pI) were calculated from these modeled three-dimensional structures using PDB2PQR.^42^ Electrostatic potential maps were computed with the programs PDB2PQR and APBS.^42,43^ Structures and surface charges were depicted using PyMOL. Prediction of signal peptide (SP) cleavage sites was performed with SignalP 6.0.^44^ Calculation of multiple amino-acid sequence alignments was performed with ClustalX 2.0.^45^

## 3 Results

### 3.1 Recombinant Expression and Purification of Apls

We overexpressed the SAPLIPs rAplE1, rAplE2, rAplK1, and rAplK2 as recombinant fusion proteins with the 6× His-tagged intein SspDnaB (Figure 1) in *E. coli*. The use of this intein enabled the efficient and specific removal of the affinity tag used for purification by immobilized-metal affinity chromatography. After affinity chromatography, the purified fusion proteins showed apparent molecular masses of ∼43 kDa as observed on Coomassie-stained SDS-PAGE gels. Additional bands of approximately 34 kDa were also visible corresponding to the 6× His-tagged intein after the splicing reaction. After an incubation step to complete the intein splicing reaction, the intensity of the lower bands appeared to be much higher and that of the upper band much lower suggesting the successful splicing of the fusion protein. Besides the shifted intensities, an additional band of approximately 7 kDa appeared corresponding to the mass of the respective recombinant Apl peptide. Further purification via Ni-NTA, by anion exchange chromatography, and by use of a reversed-phase column yielded apparently pure recombinant Apls as evidenced by SDS-PAGE (Figure 1).

### 3.2 Pore Formation Depends on the pH Value

**Figure 2:**
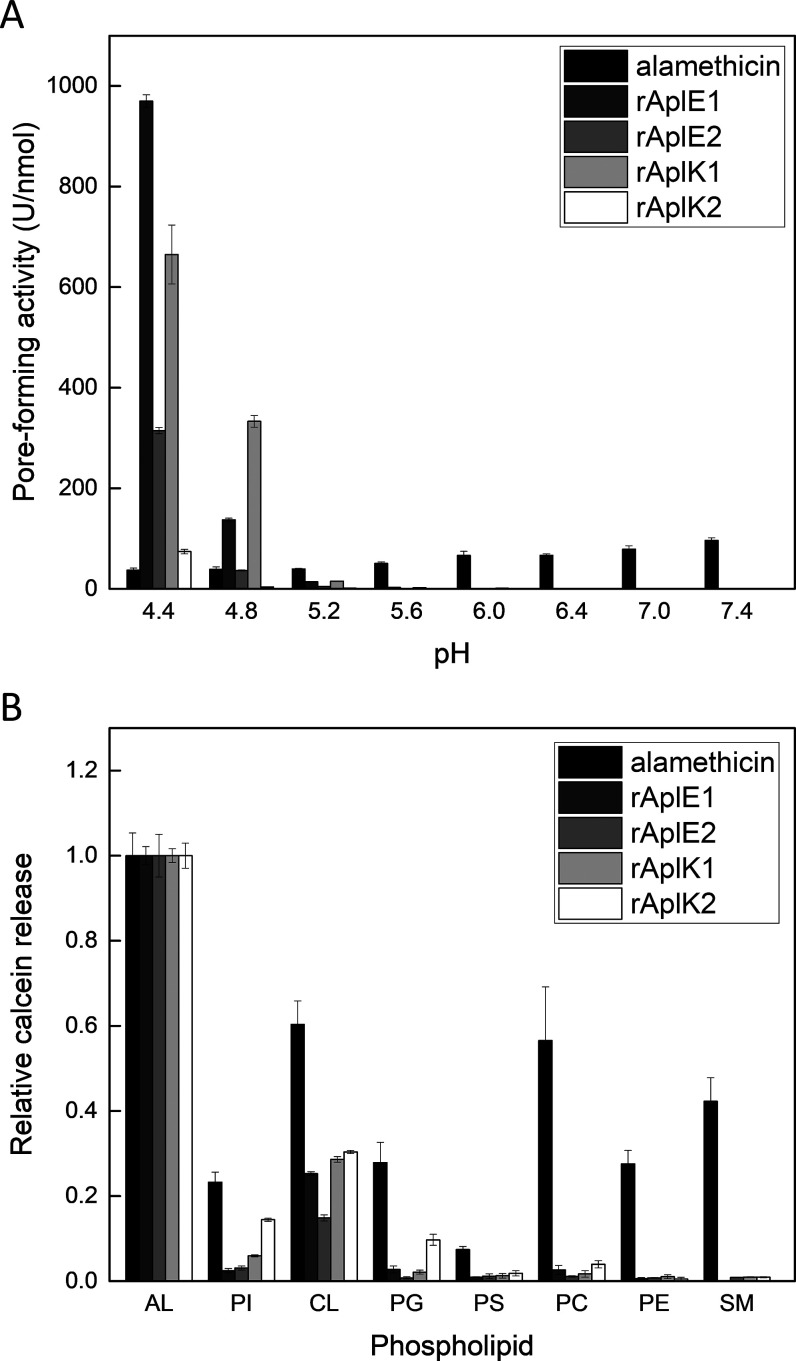
Membrane-permeabilizing activity of recombinant Apls depending on the pH value and on the lipid composition. (A) pH dependence is measured as pore-forming activity using liposomes made from AL at pH values ranging from 4.4 to 7.4. (B) Relative lipid preference of recombinant Apls tested at pH 4.4 with liposomes made from AL or PC or from 3:1 (w/w) compositions of PC, and PI, CL, PG, PS, PE, or SM. Activities with liposomes composed of AL were set to one. In (A) and in (B) alamethicin was used as a control peptide.

The pore-forming activity of recombinant Apls was investigated by monitoring the dissipation of a diffusion potential in liposomes. The depolarization of liposomes made from AL was measured at pH values ranging between 4.4 and 7.4. It became evident that the activity of the Apls studied increased dramatically with decreasing pH (Figure 2A). At a pH value of 4.4, the activities were 970 U/nmol (rAplE1), 315 U/nmol (rAplE2), 665 U/nmol (rAplK1), and 74 U/nmol (rAplK2). Conversely, increase in pH to 5.2 reduced the activity of all these Apls to less than 2% of the activity at pH 4.4. Reduction of disulfide bonds and alkylation of cysteine residues resulted in a complete loss of activity for rAplE1 and rAplK2, while the pore-forming activity of rAplE2 and rAplK1 was reduced to less than 50% as measured at pH 4.4 (Figure S1).

### 3.3 Lipid Preference of Apls Depends on Phospholipid Charge

Lipid preferences of recombinant Apls were analyzed with the calcein release assay at pH 4.4. All Apls were found to be most active with liposomes made from AL (Figure 2B). Analysis of membrane permeabilization with liposomes made from specific lipid compositions demonstrated a preference of all recombinant Apls for CL, with activities of 15–30% compared to that measured with liposomes made of AL. The protein rAplK2 showed a relative calcein release of approximately 10% with liposomes containing PG. Furthermore, a preference for PI-containing liposomes was demonstrated with rAplK1 and rAplK2 showing activities of 6% and 14% relative calcein release, respectively. Calcein release with all other Apl/lipid combinations was very low, reaching less than 5% of the level compared to the activity detected with AL (Figure 2B).

### 3.4 Antibacterial Activity of Recombinant Apls

Determination of MIC and MBC with the Gram-negative bacteria *E. coli*, *K. pneumoniae*, and *P. aeruginosa*, and with the Gram-positive bacteria *B. subtilis* and *S. aureus* showed that up to a concentration of 20 μM an antibacterial activity for any of the tested Apls could not be observed in these microdilution susceptibility assays performed at pH 5.2. For the Gram-positive *P. megaterium* only, antibacterial activity of the recombinant Apls was detectable with a minimal inhibitory and bactericidal concentration of 20 μM (Table 1).

**Table 1:** Antimicrobial Activity of AplE and AplK Proteins^a^

	MIC (MBC), μM				
	AplE1	AplE2	AplK1	AplK2	melittin
*E. coli*	>20	>20	>20	>20	0.25 (0.25)
*P. aeruginosa*	>20	>20	>20	>20	>8
*Klebsiella aerogenes*	>20	>20	>20	>20	4–8 (8)
*P. megaterium*	20 (20)	20 (20)	20 (20)	20 (20)	0.125 (0.125)
*B. subtilis*	>20	>20	>20	>20	0.0625 (0.125)
*S. aureus*	>20	>20	>20	>20	>8

a The antimicrobial activity was determined as MIC for Gram-negative and for Gram-positive bacteria. Values for MBC are shown in parentheses. Measurements were performed at pH 5.2 in duplicates with protein concentrations of up to 20 μM AplE1, AplE2, AplK1, and AplK2 and up to 8 μM melittin, respectively.

### 3.5 Recombinant Apls Permeabilize the Membrane of Live Bacteria

**Figure 3:**
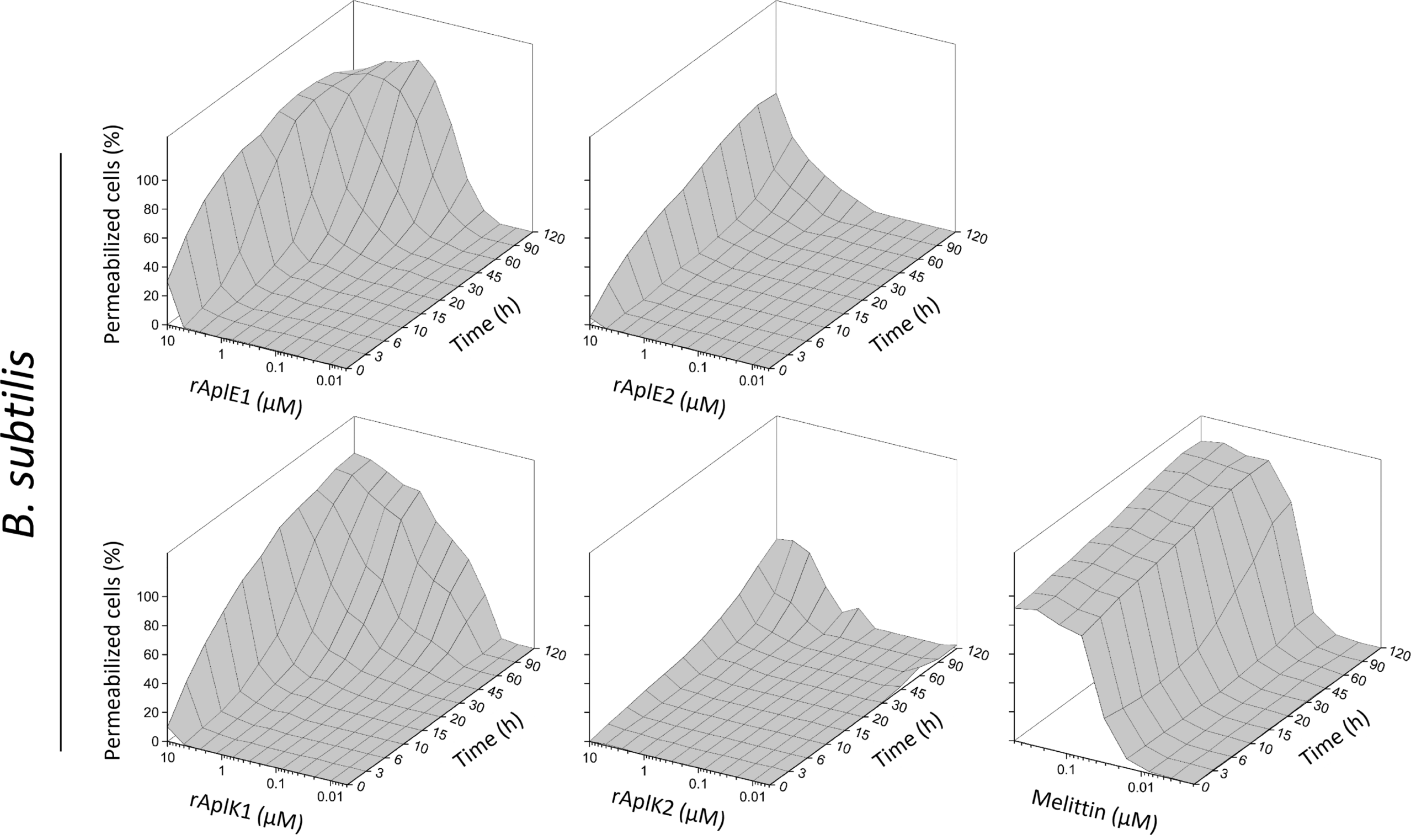
Permeabilization of live bacteria by recombinant Apls. Permeabilization of live *B. subtilis* cells was measured as an increase of the fluorescence of the DNA-intercalating dye SYTOX Green at pH 5.2. Fluorescence was followed after incubation of bacteria with serial dilutions of recombinant Apls or with melittin over time. All values were normalized to the maximum fluorescence after incubation with the control peptide melittin at 0.5 mM for 20 min. Data are from experiments performed in duplicates.

To investigate membrane permeabilization of live bacteria, we used the SYTOX Green assay. For the Gram-negative *E. coli*, no increase of fluorescence was detected with the recombinant Apls, while the control peptide melittin showed rapid permeabilization of the membrane (Figure S2). For the Gram-positive *B. subtilis*, the efficacy of membrane permeabilization appeared to be dependent on incubation period, peptide concentration, and the specific Apl. Potent membrane-permeabilizing activity was detected with rAplE1 and rAplK1, while rAplE2 and rAplK2 showed substantially lower potency (Figure 3).

### 3.6 Structural Modeling of Recombinant Apls

**Figure 4:**
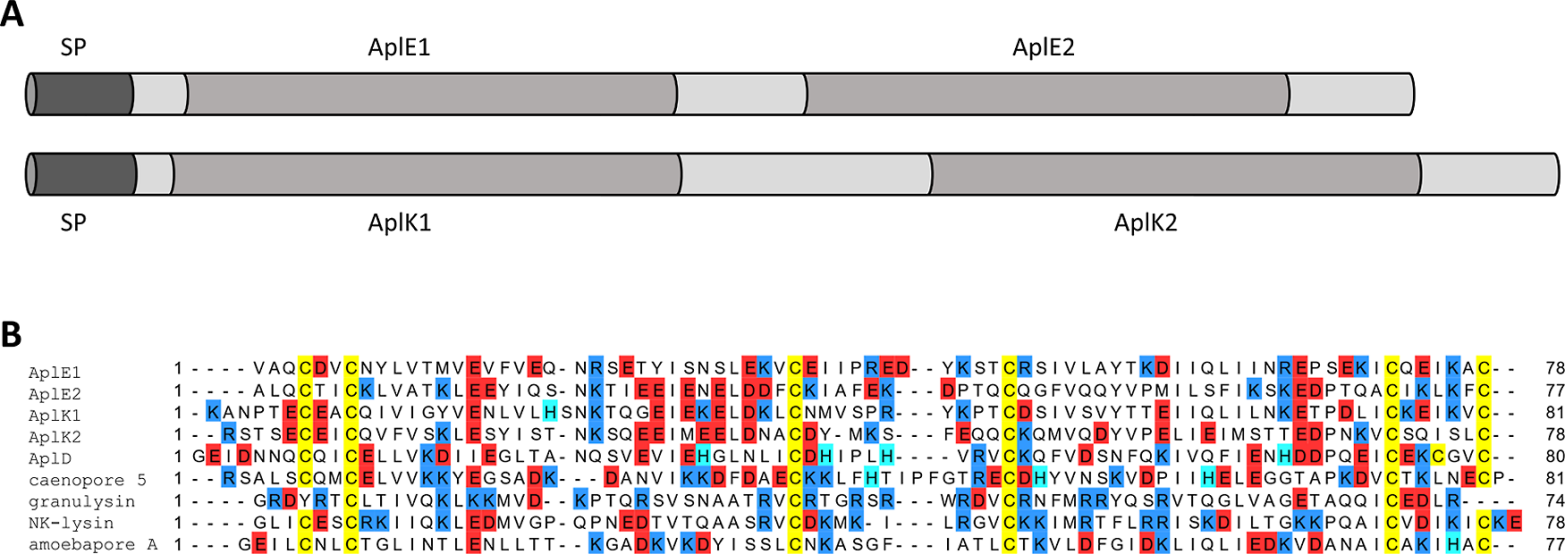
Molecular organization of the precursors AplE and AplK and comparison of the primary structure of the putative mature Apls with that of characterized antimicrobial SAPLIPs. (A) The complete precursor proteins AplE and AplK are both composed of an N-terminal SP and of two SAPLIP domains, i.e. AplE1, AplE2, AplK1, and AplK2, that are surrounded by proregions (light gray). (B) Multiple amino acid sequence alignment of separate AplE and AplK SAPLIPs, together with the already characterized AplD, caenopore 5, granulysin, NK-lysin, and amoebapore A. Arginine, and lysine residues are depicted by blue color; aspartic acid and glutamic acid residues are marked red; histidine residues are highlighted in cyan; cysteine residues are shown in yellow. Uniprot accession: AplE1/2: Q54WE4; AplK1/2: Q54WE0; AplD: Q54C60; caenopore 5: Q86FL8; granulysin: P22749; NK-lysin Q29075; amoebapore A: P34095.

Sequence analyses of the primary translation products AplE and AplK revealed that both contain a potential N-terminal SP with a length of 17 amino acid residues. Moreover, AplE and AplK are both composed of two saposin B-domains that are connected by linker regions of different length (Figure 4A).

**Figure 5:**
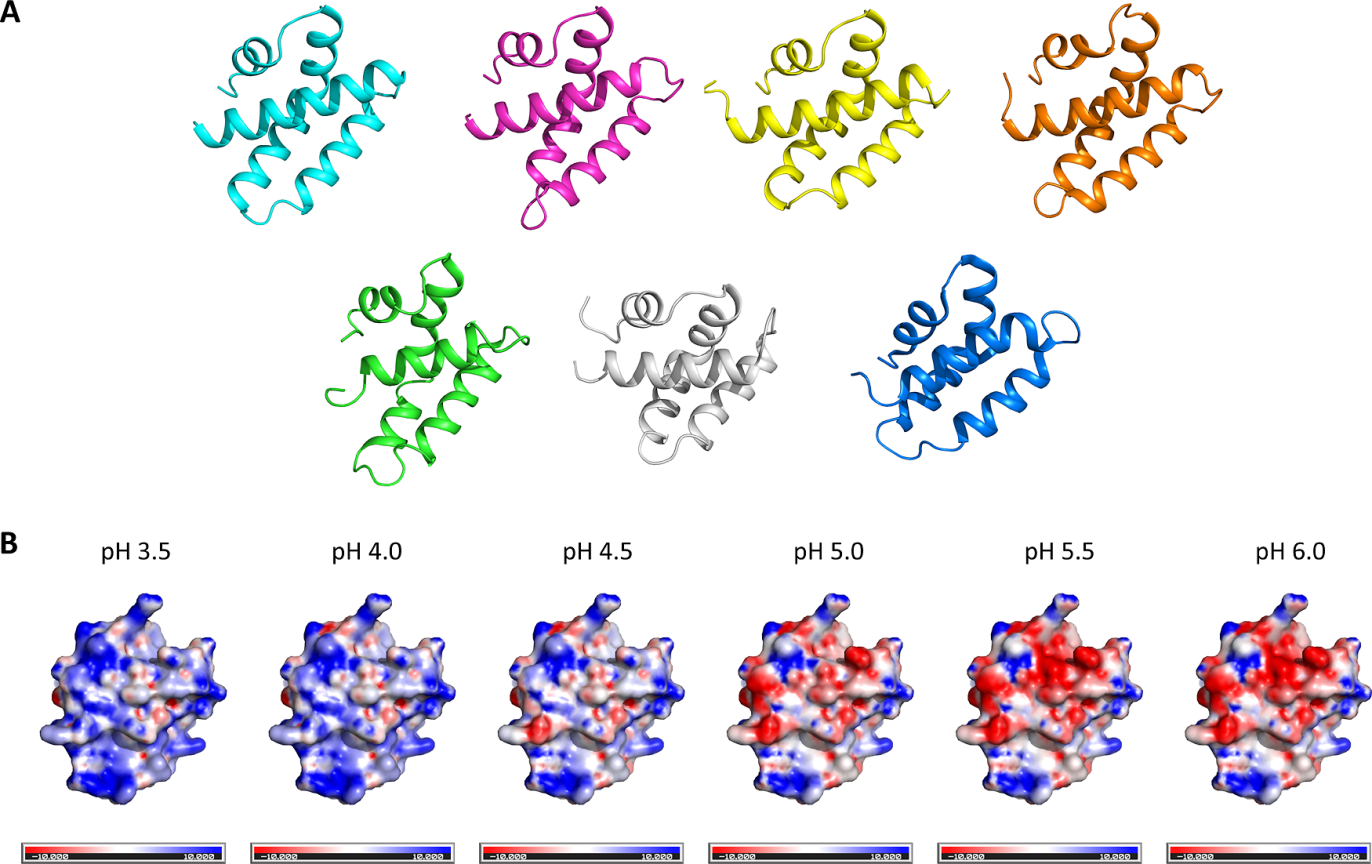
Structural comparison of recombinant Apls with characterized SAPLIP members and surface charge distribution of AplE1 at different pH values. (A) The modeled three-dimensional structures of rAplE1 (cyan), rAplE2 (magenta), rAplK1 (yellow), and rAplK2 (orange) are shown next to the solved structures of caenopore 5 (green; pdb: 2JS9), porcine NK-lysin (gray; pdb: 1NKL), and amoebapore A (blue; pdb: 1OF9) in the same orientation. The conserved array of disulfide bonds is not shown. (B) Surface charge distribution of rAplE1 within the pH range 3.5–6.0. The proportion of negative charges gradually increases with increasing pH. Negatively charged areas are colored in red, positive charges are depicted in blue (scale bar −10 to +10 *kT*/*e*). The orientation of rAplE1 is the same as in (A). In both, (A,B) helix 1 is facing the observer.

Modeling of the three-dimensional structures of the recombinant Apls and their comparison to crystal structures and NMR solution structures of characterized SAPLIP members revealed folded-leaf like structures similar to the classical SAPLIP fold with all cysteine residues forming disulfide bonds (Figure 5A). The analyzed Apls show very similar backbone structures that resemble best the structure of caenopore 5 from *C. elegans*.^19^ Accordingly, helix 1 is rotated by approximately 45° compared to helix 2 and 3, and is not in an almost orthogonal position as has been shown for NK-lysin and granulysin. In this respect, the modeled Apls differ also from the more parallel positioned helices 1, 2, and 3 of amoebapore A.^18^ A multiple amino acid sequence alignment (Figure 4B) revealed that the recombinant Apls do not only show low sequence identities with the human granulysin (14–19%), the porcine NK-lysin (18–24%), and with amoebapore A (20–27%) but also with the structurally very similar caenopore 5 (16–29%). Thus, the similarity in the three-dimensional structure of the analyzed Apls with other SAPLIPs is not mirrored by the sequence similarity.

An exemplary comparison of the molecular surface of AplE1 at different pH values revealed a gradual change in the surface charge (Figure 5B). Most of this change occurs within the relatively small range of pH 4.0–5.5.

## 4 Discussion

Here, we present the recombinant production and comprehensive biochemical characterization of four SAPLIPs from *D. discoideum*. All proteins depolarized liposomes in a pH-dependent manner; the more acidic the environment, the higher the activity, with Apls being almost inactive at pH 5.2 or above. A preference for negatively charged phospholipids, and for CL in particular, was observed. Antibacterial activity was low when determined in the classical micro susceptibility assays. However, in the SYTOX Green assay it became apparent that at least two Apls readily permeabilize bacterial membranes.

For the cellular slime mold *D. discoideum*, bioinformatic analyses revealed the existence of 17 SAPLIP encoding genes that may give rise to a total of 33 Apls.^20^ This considerable diversity and a potential interplay between different Apls or a synergistic action with other bacteriolytic factors, e.g. lysozymes,^28,29^ may result in various functions including a participation in nutrition and as part of a basal innate immunity. Different functional involvements may be mirrored by distinct transcript patterns throughout the life cycle of vegetative cells developing to the multicellular fruiting body. When we analyzed time-series transcript data of all *apl* genes using the dictyExpress site^46^ and data derived from RNA sequencing experiments,^47^ transcription of most *apl* genes was primarily found in vegetative cells and at early developmental stages, with the highest relative transcription of *aplB*, *aplC*, and *aplH* (Figure S3). In contrast, *aplD*, *aplE*, and *aplK* are virtually not transcribed in vegetative cells and show high transcript levels at the late stages of development only (Figure S4), a phenomenon that has only been emphasized for *aplD* so far.^20^ Given that at the late stages of development *D. discoideum* is starving and not feeding, it appears implausible that these Apls are involved in the digestion of food bacteria. Instead, one can speculate that these Apls may be instrumental to combat invading potential pathogens and as such are components of a defense system. In this context, the lately appearing Apls may also be considered as factors that influence the ability of the so-called sentinel cells to defend the organism against pathogenic bacteria. This special cell type comprises approximately 1% of the cells in the *D. discoideum* pseudoplasmodium and is responsible for the inactivation of toxins or pathogens by ingestion and sloughing off from the multicellular aggregates.^24^ The Apls studied here may also be functional in controlling associated microbiota while developing the multicellular fruiting body. From this perspective, a particularly interesting phenomenon are *D. discoideum* strains known as “farmers”, the spores of which carry future food bacteria. These food bacteria can regrow after spore dispersal and thus serve as food source at the new site.^48,49^ The modulation of this associated bacteria has already been reported to be dependent on lectins,^50^ a function that may be complemented by lately appearing Apls.

While transcript analyses of Apls are available, only little is known about the biochemistry of the corresponding proteins. Today, only AplD, which is organized as a single SAPLIP domain, has been purified as a recombinant protein and analyzed regarding its pore-forming, antimicrobial, and membrane-interacting characteristics.^20^ The lack of characterized SAPLIPs from *D. discoideum* and the idea of an involvement in the beginning of innate immunity put our focus on the biochemical characterization of AplE and AplK.

Both AplE and AplK represent SAPLIP members that contain two saposin B-domains (Figure 4A). The phenomenon of multiple SAPLIPs being encoded in a single precursor protein has been described not only for polypeptides from *D. discoideum*,^20^ but also for polypeptides from other species, e.g. from *C. elegans* and *N. fowleri*.^11−13^ In *N. fowleri*, the precursor protein naegleriapore B is known to be processed post-translationally into separate SAPLIPs^12^ suggesting that a similar event may also occur with the premature SAPLIPs of other organisms, i.e. *D. discoideum*. The same holds true for the name-giving saposins of vertebrates.^2,5^ As a consequence, we recombinantly produced the saposin B-domains of AplE and AplK as individual entities. These polypeptides were designated AplE1 and AplE2 derived from AplE, and AplK1 and AplK2 stemming from AplK.

We purified the Apl polypeptides as fusion proteins with the self-splicing intein SspDnaB. The Apls were then spliced from the complete fusion protein by induction of the intein through a pH and temperature shift. By this approach, we circumvented the application of proteases to remove the affinity tag that is used for purification. Major advantage of the intein is that it does not show unspecific cleavage as has been reported for enteropeptidases many times resulting in inhomogeneous or even incorrect final products.^51,52^ In addition, the use of inteins also eliminates the relatively high costs of commercially available enteropeptidases. Inteins are primarily known for being able to excise themselves from proteins, but biotechnologically modified inteins can also be used for splicing off target proteins from precursors if inserted between a tag and the protein of interest, while showing no unspecific cleavage.^53−56^ To enable efficient splicing, we inserted a glycine between SspDnaB and the Apl protein, as it has been reported earlier that the splicing velocity depends on the nature of the junction site with glycine being a good candidate for satisfying efficiency.^57^ This glycine residue remains at the N-terminus of the final polypeptide, but we do not expect any effect on protein function.

The pH dependence of the pore-forming activity of recombinant AplE and AplK SAPLIPs was pronounced. A similar pH-dependent activity has already been reported for AplD.^20^ The importance of protein charge and pH has been emphasized previously showing that highly positively charged SAPLIPs exhibit a less pH-associated behavior than those with minor positive charges.^39,58,59^ When we analyzed the AplE and AplK derived SAPLIPs, we focused on the pI value and the net charge of these polypeptides at certain pH values. The activity we monitored with the Apls studied here is in accordance with the relatively low calculated pI values of AplE1 (5.03), AplE2 (4.96), AplK1 (5.35), and AplK2 (4.17). Consequently, at pH 4.4, which represents the lowest pH value tested here, the calculated net charges of the respective polypeptides were positive for AplE1 (+3.23), AplE2 (+2.18), and AplK1 (+3.51), but negative for AplK2 (−1.37). These properties may explain why AplK2 is by far the least active of all Apls investigated so far. The very profound increase in activity within the moderate pH shift from 5.2 to 4.4 corresponds well with the change in surface charge as shown exemplarily for AplE1 in Figure 5B. Here, it becomes obvious that the change occurs mostly between pH 4.0 and 5.5.

The importance of electrostatic interaction is also reflected by the lipid preferences of the recombinant Apls. The polypeptides exerted highest activity with liposomes made from a defined lipid composition with liposomes containing CL, a lipid the headgroup of which is even dianionic.^60,61^ Furthermore, activity was only displayed with liposomes containing the monoanionic lipids PG or PI, although at a much lower level. The remarkably higher membrane-permeabilizing activity that we detected with liposomes made from AL is most likely due to the sophisticated composition of this natural lipid mixture.^62^

The generally low antibacterial activity may be explained by the low cationic state of the analyzed recombinant SAPLIPs at the pH of 5.2 used in these assays. Unfortunately, lower pH values could not be investigated as most of the tested bacteria fail to grow under these conditions. Nevertheless, expecting SAPLIPs to be found in particular in the lysosomal compartment, an in vivo function can be assumed as lysosomes in *D. discoideum* have been reported to have a pH below 3.5.^63^ Notably, membrane permeabilization of live bacteria was detected by a fluorescent dye assay, namely the SYTOX Green assay. Here, we detected activity against the Gram-positive bacterium *B. subtilis* but not against the Gram-negative representative *E. coli*. These results can be explained by assuming that the outer membrane of Gram-negative bacteria constitutes a barrier, a phenomenon that has been described several times.^20,30^

SAPLIP proteins carry out diverse functions and certainly not all are antimicrobial factors. Moreover, antimicrobial activity does not necessarily coincide with substantial pore-forming activity toward liposomes as has already been demonstrated for Naegliapores.^12^ Interestingly, SAPLIPs from the amoebae *E. histolytica* and *Entamoeba invadens* have even been described to permeabilize neither liposomes nor bacterial membranes and exert no antimicrobial activity at all.^64,65^ Instead, these polypeptides induce the fusion of lipid vesicles similar to the human saposin C.^66,67^

## 5 Conclusion

In this work, we report the recombinant production and biochemical characterization of four saposin B-domain-containing polypeptides from the social amoeba *D. discoideum*, AplE1, AplE2, AplK1, and AplK2. These polypeptides are derived from the two precursor proteins AplE and AplK, the genes of which are exclusively transcribed at the late developmental stages. The analyzed SAPLIPs readily permeabilized the membranes of live Gram-positive bacteria and are thus potential effectors of a basal innate immunity. Biochemical characterization revealed that the activity of the Apls is remarkably dependent on relatively low pH and on the presence of negatively charged phospholipids. Analysis of their surface charge emphasizes the importance of electrostatic interactions between Apls and membrane phospholipids.

## Supporting Information Available

Relative pore-forming activity of recombinant Apls after reduction and alkylation of cysteine residues (Figure S1), permeabilization of live *E. coli* by recombinant Apls (Figure S2), time-series transcript data of all *apl* genes (Figure S3), time-series transcript data of *aplE*, *aplD*, and *aplK* (Figure S4) (PDF)

## Abbreviations

AMP: antimicrobial peptide
SAPLIP: saposin-like protein
Apl: amoebapore-like peptide
LB: Luria–Bertani
TFA: trifluoroacetic acid
AL: asolectin
U: unit
PC: phosphatidylcholine
PE: phosphatidylethanolamine
PG: phosphatidylglycerol
PS: phosphatidylserine
PI: phosphatidylinositol
SM: sphingomyelin
CL: cardiolipin
MIC: minimal inhibitory concentration
MBC: minimal bactericidal concentration
CFU: colony forming units
pI: isoelectric point
SP: signal peptide

## References

[ref1] Pasupuleti M.; Schmidtchen A.; Malmsten M. Antimicrobial peptides: key components of the innate immune system. Crit. Rev. Biotechnol. 2012, 32, 143–171. 10.3109/07388551.2011.594423. 22074402

[ref2] Munford R. S.; Sheppard P. O.; O’Hara P. J. Saposin-like proteins (SAPLIP) carry out diverse functions on a common backbone structure. J. Lipid Res. 1995, 36, 1653–1663. 10.1016/S0022-2275(20)41485-3. 7595087

[ref3] Bruhn H. A short guided tour through functional and structural features of saposin-like proteins. Biochem. J. 2005, 389, 249–257. 10.1042/BJ20050051. 15992358 PMC1175101

[ref4] Kolter T.; Winau F.; Schaible U. E.; Leippe M.; Sandhoff K. Lipid-binding proteins in membrane digestion, antigen presentation, and antimicrobial defense. J. Biol. Chem. 2005, 280, 41125–41128. 10.1074/jbc.R500015200. 16230343

[ref5] O’Brien J. S.; Kishimoto Y. Saposin proteins: structure, function, and role in human lysosomal storage disorders. FASEB J. 1991, 5, 301–308. 10.1096/fasebj.5.3.2001789. 2001789

[ref6] Andersson M.; Gunne H.; Agerberth B.; Boman A.; Bergman T.; Sillard R.; Jornvall H.; Mutt V.; Olsson B.; Wigzell H. NK-lysin, a novel effector peptide of cytotoxic T and NK cells. Structure and cDNA cloning of the porcine form, induction by interleukin 2, antibacterial and antitumour activity. EMBO J. 1995, 14, 1615–1625. 10.1002/j.1460-2075.1995.tb07150.x. 7737114 PMC398254

[ref7] Peña S. V.; Hanson D. A.; Carr B. A.; Goralski T. J.; Krensky A. M. Processing, subcellular localization, and function of 519 (granulysin), a human late T cell activation molecule with homology to small, lytic, granule proteins. J. Immunol. 1997, 158, 2680–2688. 10.4049/jimmunol.158.6.2680. 9058801

[ref8] Stenger S.; Hanson D. A.; Teitelbaum R.; Dewan P.; Niazi K. R.; Froelich C. J.; Ganz T.; Thoma-Uszynski S.; Melián A.; Bogdan C.; Porcelli S. A.; Bloom B. R.; Krensky A. M.; Modlin R. L. An antimicrobial activity of cytolytic T cells mediated by granulysin. Science 1998, 282, 121–125. 10.1126/science.282.5386.121. 9756476

[ref9] Leippe M.; Ebel S.; Schoenberger O. L.; Horstmann R. D.; Müller-Eberhard H. J. Pore-forming peptide of pathogenic *Entamoeba histolytica*. Proc. Natl. Acad. Sci. U.S.A. 1991, 88, 7659–7663. 10.1073/pnas.88.17.7659. 1881907 PMC52361

[ref10] Leippe M. Amoebapores. Parasitol. Today 1997, 13, 178–183. 10.1016/S0169-4758(97)01038-7. 15275088

[ref11] Herbst R.; Ott C.; Jacobs T.; Marti T.; Marciano-Cabral F.; Leippe M. Pore-forming polypeptides of the pathogenic protozoon *Naegleria fowleri*. J. Biol. Chem. 2002, 277, 22353–22360. 10.1074/jbc.M201475200. 11948186

[ref12] Herbst R.; Marciano-Cabral F.; Leippe M. Antimicrobial and pore-forming peptides of free-living and potentially highly pathogenic *Naegleria fowleri* are released from the same precursor molecule. J. Biol. Chem. 2004, 279, 25955–25958. 10.1074/jbc.M401965200. 15075336

[ref13] Roeder T.; Stanisak M.; Gelhaus C.; Bruchhaus I.; Grötzinger J.; Leippe M. Caenopores are antimicrobial peptides in the nematode *Caenorhabditis elegans* instrumental in nutrition and immunity. Dev. Comp. Immunol. 2010, 34, 203–209. 10.1016/j.dci.2009.09.010. 19818806

[ref14] Hoeckendorf A.; Stanisak M.; Leippe M. The saposin-like protein SPP-12 is an antimicrobial polypeptide in the pharyngeal neurons of *Caenorhabditis elegans* and participates in defence against a natural bacterial pathogen. Biochem. J. 2012, 445, 205–212. 10.1042/BJ20112102. 22519640

[ref15] Hoeckendorf A.; Leippe M. SPP-3, a saposin-like protein of *Caenorhabditis elegans*, displays antimicrobial and pore-forming activity and is located in the intestine and in one head neuron. Dev. Comp. Immunol. 2012, 38, 181–186. 10.1016/j.dci.2012.05.007. 22677064

[ref16] Liepinsh E.; Andersson M.; Ruysschaert J. M.; Otting G. Saposin fold revealed by the NMR structure of NK-lysin. Nat. Struct. Biol. 1997, 4, 793–795. 10.1038/nsb1097-793. 9334742

[ref17] Anderson D. H.; Sawaya M. R.; Cascio D.; Ernst W.; Modlin R.; Krensky A.; Eisenberg D. Granulysin crystal structure and a structure-derived lytic mechanism. J. Mol. Biol. 2003, 325, 355–365. 10.1016/S0022-2836(02)01234-2. 12488100

[ref18] Hecht O.; Van Nuland N. A.; Schleinkofer K.; Dingley A. J.; Bruhn H.; Leippe M.; Grötzinger J. Solution structure of the pore-forming protein of *Entamoeba histolytica*. J. Biol. Chem. 2004, 279, 17834–17841. 10.1074/jbc.M312978200. 14970207

[ref19] Mysliwy J.; Dingley A. J.; Stanisak M.; Jung S.; Lorenzen I.; Roeder T.; Leippe M.; Grötzinger J. Caenopore-5: the three-dimensional structure of an antimicrobial protein from *Caenorhabditis elegans*. Dev. Comp. Immunol. 2010, 34, 323–330. 10.1016/j.dci.2009.11.003. 19917307

[ref20] Dhakshinamoorthy R.; Bitzhenner M.; Cosson P.; Soldati T.; Leippe M. The saposin-like protein AplD displays pore-forming activity and participates in defense against bacterial infection during a multicellular stage of *Dictyostelium discoideum*. Front. Cell. Infect. Microbiol. 2018, 8, 73 10.3389/fcimb.2018.00073. 29662839 PMC5890168

[ref21] Raper K. B. *Dictyostelium discoideum*, a new species of slime mold from decaying forest leaves. J. Agric. Res. 1935, 55, 289–316.

[ref22] Dunn J. D.; Bosmani C.; Barisch C.; Raykov L.; Lefrançois L. H.; Cardenal-Muñoz E.; López-Jiménez A. T.; Soldati T. Eat prey, live: *Dictyostelium discoideum* as a model for cell-autonomous defenses. Front. Immunol. 2018, 8, 1906 10.3389/fimmu.2017.01906. 29354124 PMC5758549

[ref23] Steinert M.; Leippe M.; Roeder T. Surrogate hosts: protozoa and invertebrates as models for studying pathogen-host interactions. Int. J. Med. Microbiol. 2003, 293, 321–332. 10.1078/1438-4221-00275. 14695060

[ref24] Chen G.; Zhuchenko O.; Kuspa A. Immune-like phagocyte activity in the social amoeba. Science 2007, 317, 678–681. 10.1126/science.1143991. 17673666 PMC3291017

[ref25] Kin K.; Schaap P. Evolution of multicellular complexity in the dictyostelid social amoebas. Genes 2021, 12, 487 10.3390/genes12040487. 33801615 PMC8067170

[ref26] Martín-González J.; Montero-Bullón J.; Lacal J. *Dictyostelium discoideum* as a non-mammalian biomedical model. Microb. Biotechnol. 2021, 14, 111–125. 10.1111/1751-7915.13692. 33124755 PMC7888446

[ref27] Schaap P. Evolution of size and pattern in the social amoebas. Bioessays 2007, 29, 635–644. 10.1002/bies.20599. 17563079 PMC3045520

[ref28] Müller I.; Šubert N.; Otto H.; Herbst R.; Rühling H.; Maniak M.; Leippe M. A Dictyostelium mutant with reduced lysozyme levels compensates by increased phagocytic activity. J. Biol. Chem. 2005, 280, 10435–10443. 10.1074/jbc.M411445200. 15640146

[ref29] Lamrabet O.; Jauslin T.; Lima W. C.; Leippe M.; Cosson P. The multifarious lysozyme arsenal of *Dictyostelium discoideum*. Dev. Comp. Immunol. 2020, 107, 103645 10.1016/j.dci.2020.103645. 32061941

[ref30] Guilhen C.; Lima W. C.; Ifrid E.; Crespo-Yañez X.; Lamrabet O.; Cosson P. A new family of bacteriolytic proteins in *Dictyostelium discoideum*. Front. Cell. Infect. Microbiol. 2021, 10, 617310 10.3389/fcimb.2020.617310. 33614529 PMC7886984

[ref31] Munford R. S.; Hunter J. P. Acyloxyacyl hydrolase, a leukocyte enzyme that deacylates bacterial lipopolysaccharides, has phospholipase, lysophospholipase, diacylglycerollipase, and acyltransferase activities in vitro. J. Biol. Chem. 1992, 267, 10116–10121. 10.1016/S0021-9258(19)50207-1. 1577781

[ref32] Jauslin T.; Lamrabet O.; Crespo-Yañez X.; Marchetti A.; Ayadi I.; Ifrid E.; Guilhen C.; Leippe M.; Cosson P. How phagocytic cells kill different bacteria: a quantitative analysis using *Dictyostelium discoideum*. mBio 2021, 12, e03169-20 10.1128/mbio.03169-20. 33593980 PMC8545105

[ref33] Brock D. A.; Gomer R. H. A cell-counting factor regulating structure size in *Dictyostelium*. Genes Dev. 1999, 13, 1960–1969. 10.1101/gad.13.15.1960. 10444594 PMC316923

[ref34] Okuwa T.; Katayama T.; Takano A.; Kodaira K.; Yasukawa H. Two cell-counting factors regulate the aggregate size of the cellular slime mold *Dictyostelium discoideum*. Dev. Growth Differ. 2001, 43, 735–744. 10.1046/j.1440-169X.2001.00615.x. 11737154

[ref35] Ayadi I.; Lamrabet O.; Munoz-Ruiz R.; Jauslin T.; Guilhen C.; Cosson P. Extracellular and intracellular destruction of *Pseudomonas aeruginosa* by *Dictyostelium discoideum* phagocytes mobilize different antibacterial mechanisms. Mol. Microbiol. 2024, 121, 69–84. 10.1111/mmi.15197. 38017607

[ref36] Lobstein J.; Emrich C. A.; Jeans C.; Faulkner M.; Riggs P.; Berkmen M. SHuffle, a novel *Escherichia coli* protein expression strain capable of correctly folding disulfide bonded proteins in its cytoplasm. Microb. Cell Factories 2012, 11, 753 10.1186/1475-2859-11-56. PMC3526497 22569138

[ref37] Amaranto M.; Vaccarello P.; Correa E. M. E.; Barra J. L.; Godino A. Novel intein-based self-cleaving affinity tag for recombinant protein production in *Escherichia coli*. J. Biotechnol. 2021, 332, 126–134. 10.1016/j.jbiotec.2021.04.003. 33878389

[ref38] Loew L. M.; Rosenberg I.; Bridge M.; Gitler C. Diffusion potential cascade. Convenient detection of transferable membrane pores. Biochemistry 1983, 22, 837–844. 10.1021/bi00273a020. 6838828

[ref39] Bruhn H.; Riekens B.; Berninghausen O.; Leippe M. Amoebapores and NK-lysin, members of a class of structurally distinct antimicrobial and cytolytic peptides from protozoa and mammals: a comparative functional analysis. Biochem. J. 2003, 375, 737–744. 10.1042/bj20030250. 12917014 PMC1223731

[ref40] Andrä J.; Herbst R.; Leippe M. Amoebapores, archaic effector peptides of protozoan origin, are discharged into phagosomes and kill bacteria by permeabilizing their membranes. Dev. Comp. Immunol. 2003, 27, 291–304. 10.1016/S0145-305X(02)00106-4. 12590963

[ref41] Mirdita M.; Schütze K.; Moriwaki Y.; Heo L.; Ovchinnikov S.; Steinegger M. ColabFold: making protein folding accessible to all. Nat. Methods 2022, 19, 679–682. 10.1038/s41592-022-01488-1. 35637307 PMC9184281

[ref42] Dolinsky T. J.; Czodrowski P.; Li H.; Nielsen J. E.; Jensen J. H.; Klebe G.; Baker N. A. PDB2PQR: expanding and upgrading automated preparation of biomolecular structures for molecular simulations. Nucleic Acids Res. 2007, 35, W522–W525. 10.1093/nar/gkm276. 17488841 PMC1933214

[ref43] Jurrus E.; Engel D.; Star K.; Monson K.; Brandi J.; Felberg L. E.; Brookes D. H.; Wilson L.; Chen J.; Liles K.; Chun M.; Li P.; Gohara D. W.; Dolinsky T.; Konecny R.; Koes D. R.; Nielsen J. E.; Head-Gordon T.; Geng W.; Krasny R.; Wei G. W.; Holst M. J.; McCammon J. A.; Baker N. A. Improvements to the APBS biomolecular solvation software suite. Protein Sci. 2018, 27, 112–128. 10.1002/pro.3280. 28836357 PMC5734301

[ref44] Teufel F.; Almagro Armenteros J. J.; Johansen A. R.; Gíslason M. H.; Pihl S. I.; Tsirigos K. D.; Winther O.; Brunak S.; von Heijne G.; Nielsen H. SignalP 6.0 predicts all five types of signal peptides using protein language models. Nat. Biotechnol. 2022, 40, 1023–1025. 10.1038/s41587-021-01156-3. 34980915 PMC9287161

[ref45] Larkin M. A.; Blackshields G.; Brown N. P.; Chenna R.; McGettigan P. A.; McWilliam H.; Valentin F.; Wallace I. M.; Wilm A.; Lopez R.; Thompson J. D.; Gibson T. J.; Higgins D. G. Clustal W and Clustal X version 2.0. Bioinformatics 2007, 23, 2947–2948. 10.1093/bioinformatics/btm404. 17846036

[ref46] Stajdohar M.; Rosengarten R. D.; Kokosar J.; Jeran L.; Blenkus D.; Shaulsky G.; Zupan B. dictyExpress: a web-based platform for sequence data management and analytics in *Dictyostelium* and beyond. BMC Bioinf. 2017, 18, 291 10.1186/s12859-017-1706-9. PMC5457571 28578698

[ref47] Katoh-Kurasawa M.; Hrovatin K.; Hirose S.; Webb A.; Ho H. I.; Zupan B.; Shaulsky G. Transcriptional milestones in *Dictyostelium* development. Genome Res. 2021, 31, 1498–1511. 10.1101/gr.275496.121. 34183452 PMC8327917

[ref48] Brock D. A.; Douglas T. E.; Queller D. C.; Strassmann J. E. Primitive agriculture in a social amoeba. Nature 2011, 469, 393–396. 10.1038/nature09668. 21248849

[ref49] Brock D. A.; Read S.; Bozhchenko A.; Queller D. C.; Strassmann J. E. Social amoeba farmers carry defensive symbionts to protect and privatize their crops. Nat. Commun. 2013, 4, 2385 10.1038/ncomms3385. 24029835

[ref50] Dinh C.; Farinholt T.; Hirose S.; Zhuchenko O.; Kuspa A. Lectins modulate the microbiota of social amoebae. Science 2018, 361, 402–406. 10.1126/science.aat2058. 30049880

[ref51] Wearne S. J. Factor Xa cleavage of fusion proteins. Elimination of non-specific cleavage by reversible acylation. FEBS Lett. 1990, 263, 23–26. 10.1016/0014-5793(90)80696-G. 2185034

[ref52] Shahravan S. H.; Qu X.; Chan I. S.; Shin J. A. Enhancing the specificity of the enterokinase cleavage reaction to promote efficient cleavage of a fusion tag. Protein Expr. Purif. 2008, 59, 314–319. 10.1016/j.pep.2008.02.015. 18406169 PMC2441903

[ref53] Vila-Perelló M.; Muir T. W. Biological applications of protein splicing. Cell 2010, 143, 191–200. 10.1016/j.cell.2010.09.031. 20946979 PMC3004290

[ref54] Shah N. H.; Muir T. W. Inteins: nature’s gift to protein chemists. Chem. Sci. 2014, 5, 446–461. 10.1039/C3SC52951G. 24634716 PMC3949740

[ref55] Prabhala S. V.; Gierach I.; Wood D. W. The evolution of intein-based affinity methods as reflected in 30 years of patent history. Front. Mol. Biosci. 2022, 9, 857566 10.3389/fmolb.2022.857566. 35463948 PMC9033041

[ref56] Lahiry A.; Fan Y.; Stimple S. D.; Raith M.; Wood D. W. Inteins as tools for tagless and traceless protein purification. J. Chem. Technol. Biotechnol. 2018, 93, 1827–1835. 10.1002/jctb.5415.

[ref57] Mathys S.; Evans T. C.; Chute I. C.; Wu H.; Chong S.; Benner J.; Liu X. Q.; Xu M. Q. Characterization of a self-splicing mini-intein and its conversion into autocatalytic N- and C-terminal cleavage elements: facile production of protein building blocks for protein ligation. Gene 1999, 231, 1–13. 10.1016/S0378-1119(99)00103-1. 10231563

[ref58] Bruhn H.; Leippe M. Comparative modeling of amoebapores and granulysin based on the NK-lysin structure - structural and functional implications. Biol. Chem. 1999, 380, 1001–1007. 10.1515/bc.1999.124. 10494853

[ref59] Ortjohann M.; Leippe M. Characterization of NK-lysin A, a potent antimicrobial peptide from the zebrafish *Danio rerio*. Dev. Comp. Immunol. 2025, 162, 105266 10.1016/j.dci.2024.105266. 39303911

[ref60] Olofsson G.; Sparr E. Ionization constants pKa of cardiolipin. PLoS One 2013, 8, e73040 10.1371/journal.pone.0073040. 24058458 PMC3772843

[ref61] Sathappa M.; Alder N. N. The ionization properties of cardiolipin and its variants in model bilayers. Biochim. Biophys. Acta 2016, 1858, 1362–1372. 10.1016/j.bbamem.2016.03.007. 26965987 PMC4897776

[ref62] Johns A.; Morris S.; Edwards K.; Quirino R. L. Asolectin from soybeans as a natural compatibilizer for cellulose-reinforced biocomposites from tung oil. J. Appl. Polym. Sci. 2015, 132, 41833 10.1002/app.41833.

[ref63] Marchetti A.; Lelong E.; Cosson P. A measure of endosomal pH by flow cytometry in *Dictyostelium*. BMC Res. Notes 2009, 2, 7 10.1186/1756-0500-2-7. 19138423 PMC2632630

[ref64] Winkelmann J.; Leippe M.; Bruhn H. A novel saposin-like protein of *Entamoeba histolytica* with membrane-fusogenic activity. Mol. Biochem. Parasitol. 2006, 147, 85–94. 10.1016/j.molbiopara.2006.01.010. 16529828

[ref65] Michalek M.; Leippe M. Mechanistic insights into the lipid interaction of an ancient saposin-like protein. Biochemistry 2015, 54, 1778–1786. 10.1021/acs.biochem.5b00094. 25715682

[ref66] Vaccaro A. M.; Tatti M.; Ciaffoni F.; Salvioli R.; Serafino A.; Barca A. Saposin C induces pH-dependent destabilization and fusion of phosphatidylserine-containing vesicles. FEBS Lett. 1994, 349, 181–186. 10.1016/0014-5793(94)00659-8. 8050562

[ref67] Qi X.; Chu Z. Fusogenic domain and lysines in saposin C. Arch. Biochem. Biophys. 2004, 424, 210–218. 10.1016/j.abb.2004.02.023. 15047193

