# Supplementary material for: Molecular Characterization of Ancient Prosaposin-like Proteins from the Protist Dictyostelium discoideum
Source: Biochemistry. 2024 Oct 18;63(21):2768–77. doi: 10.1021/acs.biochem.4c00479 (PMC11542183; doi:10.1021/acs.biochem.4c00479)
Supplement: Supplementary file 1 — bi4c00479_si_001.pdf [file bi4c00479_si_001.pdf]

Supporting Information

Molecular characterization of ancient prosaposin-like proteins from the protist *Dictyostelium discoideum*

Marius Ortjohann[1] and Matthias Leippe[1]*

[1]Comparative Immunobiology, Zoological Institute, Christian-Albrechts-Universität Kiel, Am Botanischen Garten 1-9; D-24118 Kiel, Germany

*Corresponding author.

Prof. Dr. Matthias Leippe, E-Mail: mleippe@zoologie.uni-kiel.de

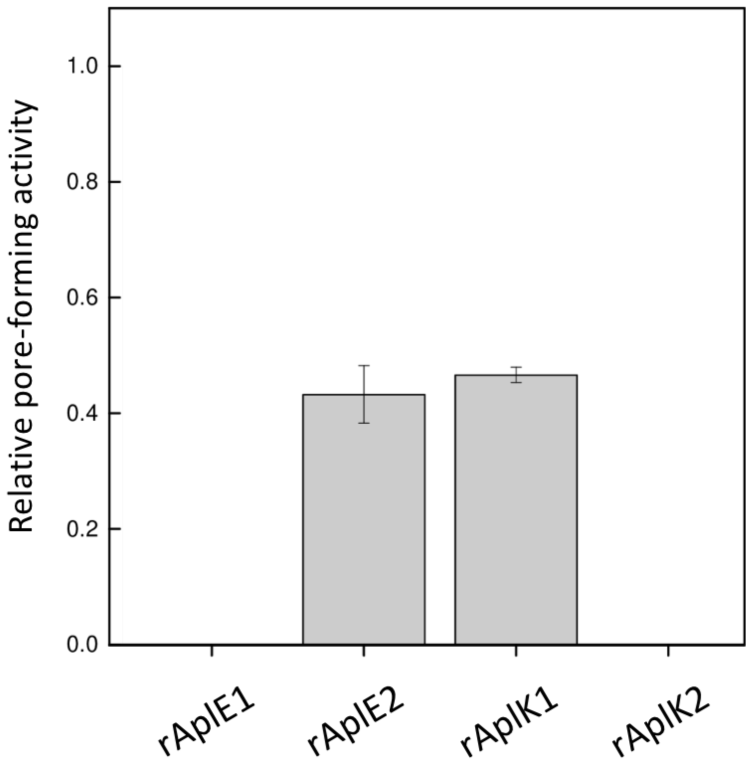

Figure S1: Relative pore-forming activity of recombinant Apls after reduction and alkylation of cysteine residues. Liposome-depolarizing activity of rAplE1, rAplE2, rAplK1, and rAplK2 after reduction and alkylation of cysteine residues with DTT and IAA are shown in comparison to the untreated polypeptides, which are set to one. Activity was tested at pH 4.4. with liposomes made from asolectin.

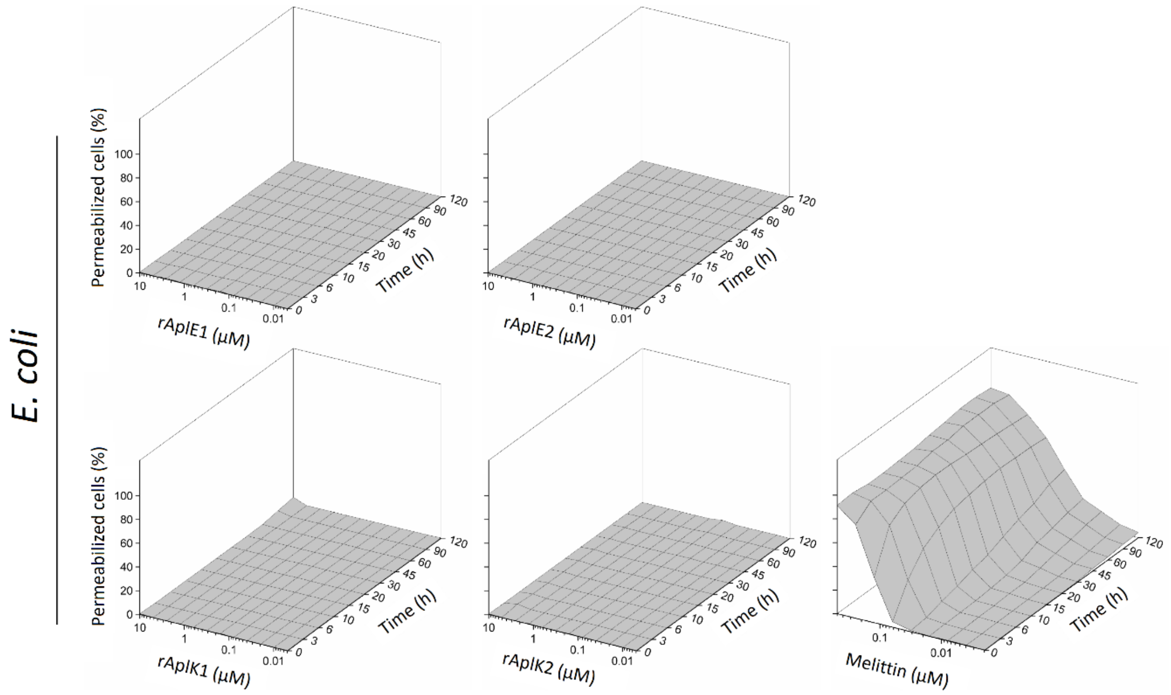

Figure S2: Permeabilization of live *E. coli* by recombinant Apls. Permeabilization of live bacterial cells was measured as an increase of the fluorescence of the DNA-intercalating dye SYTOX green at pH 5.2. Fluorescence was followed after incubation of cells with serial dilutions of recombinant Apls or with melittin over time. All values were normalized to the maximum fluorescence after incubation with the control peptide melittin at 0.5 mM for 20 min. Data are from experiments performed in dublicates.

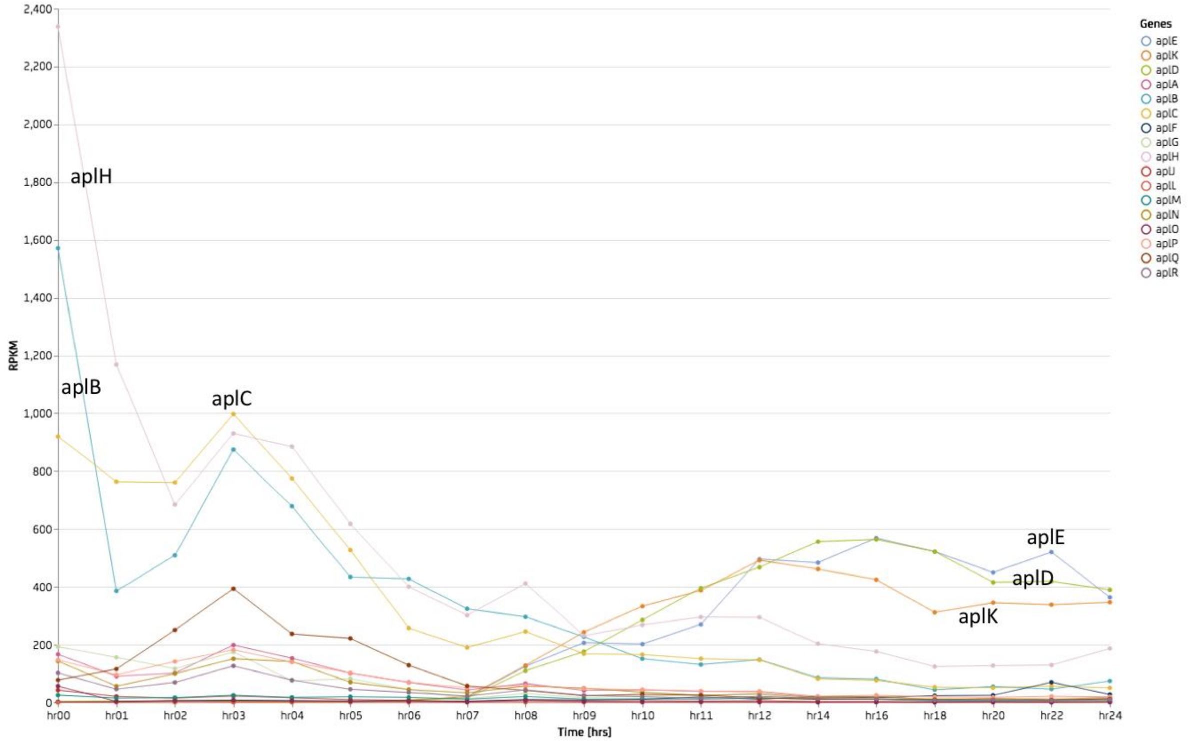

Figure S3: Time-series transcript data of all *apl* genes. RNA sequencing data are derived from Katoh-Kurasawa et al., 2021 and analyzed using the dictyExpress site (Stajdohar et al., 2017). Transcription profiles were measured at the indicated time points as Reads Per Kilobase Million (RPKM). The highest transcribed genes at the early stages of *D. discoideum* (*aplH*, *aplB*, and *aplC*) and those at the late stages (*aplE*, *aplD*, and *aplK*) are labelled.

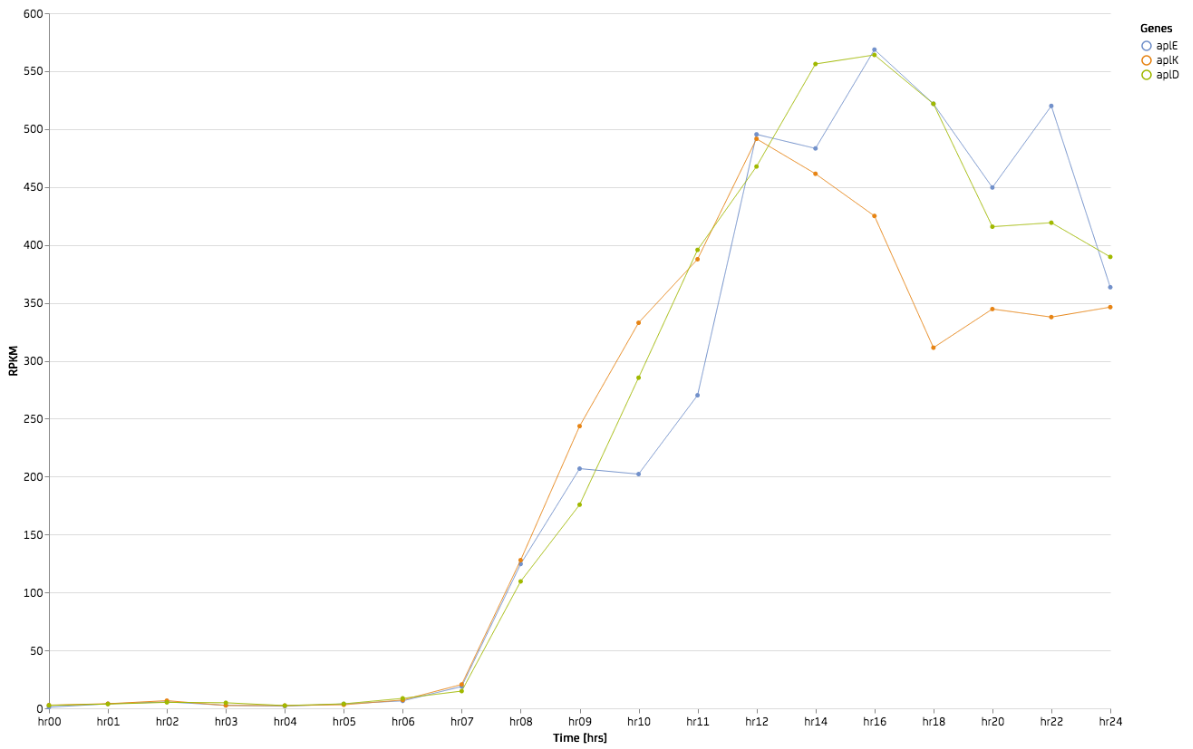

Figure S4: Time-series transcript data of the selected *aplE*, *aplD*, and *aplK*. RNA sequencing data are derived from Katoh-Kurasawa et al., 2021 and analyzed using the dictyExpress site (Stajdohar et al., 2017). Transcription profiles were measured at the indicated time points as Reads Per Kilobase Million (RPKM).

Katoh-Kurasawa, M., Hrovatin, K., Hirose, S., Webb, A., Ho, H.I., Zupan, B., Shaulsky, G., 2021. Transcriptional milestones in Dictyostelium development. Genome Res. 31, 1498-511.

Stajdohar, M., Rosengarten, R.D., Kokosar, J., Jeran, L., Blenkus, D., Shaulsky, G., Zupan, B., 2017. dictyExpress: a web-based platform for sequence data management and analytics in *Dictyostelium* and beyond. BMC Bioinformatics. 18, 291.
